# Physical Activity in Older Men: Longitudinal Associations with Inflammatory and Hemostatic Biomarkers, N-Terminal Pro-Brain Natriuretic Peptide, and Onset of Coronary Heart Disease and Mortality

**DOI:** 10.1111/jgs.12748

**Published:** 2014-03-17

**Authors:** Barbara J Jefferis, Peter H Whincup, Lucy T Lennon, Olia Papacosta, S Goya Wannamethee

**Affiliations:** * Department of Primary Care and Population Health, University College LondonLondon, UK; † Population Health Domain Physical Activity Research Group, University College LondonLondon, UK; ‡ Division of Population Health Sciences and Education, St George's, University of LondonLondon, UK

**Keywords:** physical activity, CHD, inflammation, NT-proBNP, older adults, prospective cohort

## Abstract

**Objectives:**

To examine associations between habitual physical activity (PA) and changes in PA and onset of coronary heart disease (CHD) and the pathways linking PA to CHD.

**Design:**

British Regional Heart Study population-based cohort; men completed questionnaires in 1996 and 1998 to 2000, attended rescreen in 1998 to 2000, and were followed up to June 2010.

**Setting:**

Community.

**Participants:**

Of 4,252 men recruited from primary care centers (77% of those invited and eligible) who were rescreened in 1998 to 2000, 3,320 were ambulatory and free from CHD, stroke, and heart failure and participated in the current study.

**Measurements:**

Usual PA (regular walking and cycling, recreational activity and sport). Outcome was first fatal or nonfatal myocardial infarction.

**Results:**

In 3,320 ambulatory men, 303 first and 184 fatal CHD events occurred during a median of 11 years of follow-up; 9% reported no usual PA, 23% occasional PA, and 68% light or more-intense PA. PA was inversely associated with novel risk markers C-reactive protein, D-dimer, von Willebrand Factor and N-terminal pro-brain natriuretic peptide (NT-proBNP). Compared with no usual PA, hazard ratios (HRs) for CHD events, adjusted for age and region, were 0.52 (95% confidence interval (CI) = 0.34–0.79) for occasional PA, 0.47 (95% CI = 0.30–0.74) for light PA, 0.51 (95% CI = 0.32–0.82) for moderate PA, and 0.44 (95% CI = 0.29–0.65) for moderately vigorous or vigorous PA (*P* for linear trend = .004). Adjustment for established and novel risk markers somewhat attenuated HRs and abolished linear trends. Compared with men who remained inactive, men who maintained at least light PA had an HR for CHD events of 0.73 (95% CI = 0.53–1.02) and men whose PA level increased had an HR of 0.86 (95% CI = 0.55–1.35).

**Conclusion:**

Even light PA was associated with significantly lower risk of CHD events in healthy older men, partly through inflammatory and hemostatic mechanisms and cardiac function (NT-proBNP).

Coronary heart disease (CHD) rates rise rapidly with age, so there is a pressing need to understand the effect of modifiable behaviors such as physical activity (PA) for primary prevention of CHD in older adults. This is particularly important because PA declines to low levels in older age,^1^ and (in contrast with middle age) the quantity and intensity of PA required for primary prevention of CHD in older adults remains unclear.^2^ A recent meta-analysis concluded that the most-active adults were at 30% to 35% lower risk of CHD and that those who were moderately active were at 20% to 25% lower risk of developing CHD than those who were the least active,^2^ but data on older adults are sparse. Only two of the 30 studies included participants with median age of 65 and older at baseline, yet in the INTERHEART study, PA was found to protect against CHD more strongly in older than in younger men.^3^ Furthermore, in older age, the role of established (blood pressure, lipids, adiposity) and novel risk factors as mediators between PA and CHD is not clear. C-reactive protein (CRP), a marker of inflammation that is particularly strongly linked to fatal CHD in older adults,^4^ von Willebrand factor (vWF), a marker of endothelial dysfunction; D-dimer, a marker of fibrinolytic activity; and N-terminal pro-brain natriuretic peptide (NT-proBNP), a marker of cardiac injury, are all associated with PA levels^5^–^8^ and prospectively associated with CHD^9^–^11^ or cardiovascular disease (CVD)^6^ and heart failure^12^ in healthy older adults. Given that levels of these markers rise with age, they may be important mediators between PA and CHD risk at older ages. There is some evidence that inflammation is an important mediator between PA and composite end points (including myocardial infarction (MI), coronary revascularization, stroke, and heart failure),^13^,^14^ but the mediating role of other markers is less clear. Associations between habitual PA and onset of CHD in healthy community-dwelling older men, whether changes in PA levels in later life are associated with onset of CHD, and the pathways linking PA to CHD were therefore examined. All-cause mortality was also examined as an end point.

## Methods

The British Regional Heart Study is a prospective cohort of 7,735 men aged 40 to 59 in 1978 to 1980 recruited from a single primary care center in each of 24 British towns. Men were followed up for cardiovascular morbidity and all-cause mortality. At age 56 to 74 (in 1996), men completed a postal questionnaire that collected detailed medical history and social, demographic, and health behavior data. At age 60 to 79 (in 1998–2000), 4,252 participants attended for follow-up measurements (77% response rate)^15^ and completed further questionnaires. Nurses took anthropometric measures and an electrocardiogram (including resting heart rate) and recorded blood pressure and lung function (forced vital capacity (FVC) and forced expiratory volume in 1 second (FEV_1_)).^15^ Chronic obstructive airway disease was defined as a ratio of FEV_1_ to FVC of less than 0.70. Fasting venous blood samples were collected and analyzed for lipids, vitamin C, and creatinine.^16^,^17^ Estimated glomerular filtration rate (eGFR) was estimated from serum creatinine using the Modification of Diet in Renal Disease equation,^18^ and participants with an eGFR less than 60 were categorized as having chronic kidney disease. Plasma levels of D-dimer and vWF antigen were measured using enzyme-linked immunosorbent assays, and CRP was assayed using ultrasensitive nephelometry. NT-proBNP was measured using the electrochemiluminescence method.^6^ Presence of depression was identified according to current antidepressant use reported in questionnaires (British National Formulary Code 4.3). Preexisting physician-diagnosed CHD, stroke, or diabetes mellitus (DM) was identified from questionnaire data and general practitioner records according to whether men reported that a doctor had ever told them that they had angina pectoris or MI (heart attack, coronary thrombosis), stroke, “other heart trouble,” or DM in 1998 to 2000 or had had a major nonfatal MI or stroke event or been diagnosed with DM, based on the regular surveillance of general practitioner's records. Prevalent DM was defined as doctor diagnosis of DM or fasting glucose of 7 mmol/L or greater (World Health Organization (WHO) criteria) at the 1998 to 2000 examination. Nine hundred twenty-eight individuals with a history of doctor-diagnosed CHD, stroke, or heart failure and five nonambulatory individuals were excluded to reduce the risk of reverse causality. All relevant local research ethics committees provided ethical approval, and all men provided informed written consent to the investigation.

### Physical Activity

In 1996 and in 1998 to 2000, men self-reported usual PA under the headings of regular walking or cycling, recreational activity, and sporting (vigorous) activity. Regular walking and cycling related to weekday journeys that included travel to and from work. Recreational activity included gardening, pleasure walking, and do-it-yourself jobs. Sporting activity included running, golf, swimming, tennis and sailing. A PA score (validated in relation to heart rate and FEV_1_^5^,^19^) was derived for each man. Scores were assigned for each type of activity and duration on the basis of the intensity and energy demands of the activities reported based on Minnesota intensity codes. Men were categorized into six groups based on their total score: inactive (0–2); occasional (3–5), regular walking or recreational activity only; light (6–8), more frequent recreational activities, sporting exercise less than once per week, or regular walking plus some recreational activity; moderate (9–12), cycling or very frequent weekend recreational activities plus regular walking or sporting activity once per week; moderately vigorous (13–20), sporting activity at least once per week or frequent cycling plus frequent recreational activities or walking or frequent sporting activities only; and vigorous (>21), very frequent sporting exercise or frequent sporting exercise plus other recreational activities. The moderately vigorous and vigorous groups were combined because of low numbers of CHD events in these groups. The total score for each man is not a measure of total time spent doing PA but a relative measure of how much PA has been performed.

### CHD Mortality and Morbidity

All men were followed up for all-cause mortality and first fatal or nonfatal MI occurring between the 1998 to 2000 survey and June 2010. All deaths were ascertained through the National Health Services Central Registers, MI deaths were those with death certificates with *International Classification of Disease* (ICD), Ninth Revision, codes 410 to 414 and ICD, Tenth Revision, codes I21 to I23 and I252. Nonfatal events were recorded from reviews every 2 years of primary care notes (which include correspondence and diagnoses from secondary care).^15^ MI was reported as heart attack or coronary thrombosis, diagnosed in accordance with WHO criteria.^20^

### Statistical Methods

Means, medians, or proportions of behavioral and demographic factors selected a priori were calculated according to usual PA level in 1998 to 2000. Trends across the five PA categories were tested using linear regression models for continuous variables and chi-square tests for categorical variables.

Cox regression models were used to estimate associations between 1998 to 2000 PA score and CHD risk or all-cause mortality. Survival times were censored at date of MI, death from any cause, or end of follow-up period, whichever occurred first. The time origin was date of examination in 1998 to 2000. The HRs for categories of PA in 1998 to 2000 were estimated, and then trends were tested using the continuous PA score, adjusted for age and region of residence. Models were adjusted for covariates associated with CHD risk and PA: first established social and behavioral risk factors, then biological risk markers, then markers of inflammation and coagulation (CRP, vWF, D-dimer), and then NT-proBNP were added sequentially. In sensitivity analyses, the first 2 years of follow-up were excluded. Population attributable risk percentage was calculated as (P_e_ (RR_e_ − 1)/[1 + P_e_ (RR_e_ − 1)]) × 100 using the hazard ratio (HR) comparing none or occasional PA with light or more as RR_e_ and the prevalence of no or occasional activity as P_e_.

To investigate changes in PA levels between 1996 and 1998 to 2000, the lowest two categories (none and occasional activity) were compared with the highest four (light through vigorous activity). Men in the lower PA groups walked regularly or participated in some recreational activity, in the more-active groups (from light upward), they participated in these activities more frequently, as well as some kind of sporting exercise. Means, medians, or proportions of behavioral and demographic factors selected a priori were calculated according to change in PA level. Cox regression models were used to estimate associations between change in PA level from 1996 to 1998 to 2000 and risk of CHD or mortality, following the same analysis plan as for PA in 1998 to 2000.

## Results

In 1998 to 2000, of 4,252 men aged 60 to 79 who attended their primary care center for rescreen as part of the established British Regional Heart Study,^15^ 4,097 (96%) reported complete questionnaire data on usual PA levels. Nine hundred twenty-eight participants with a history (self-report of physician diagnosis or medical record) of CHD, stroke, or heart failure and a further five participants who used a wheelchair were excluded, leaving an analysis sample of 3,320 men. Of the 3,320 men, mean age at rescreen (1998–2000) was 68.3 ± 5.4. There were 303 first (fatal or nonfatal) CHD events during a median follow-up of 10.9 years (32,070 person-years, 9.4 cases per 1,000 person-years), to June 2010, and 184 fatal CHD events during a median follow-up of 11.0 years (32,712 person-years, 5.6 cases per 1,000 person-years).

### PA Levels and Their Correlates

The prevalence of PA is reported in Table 1. More-active men were more likely to be from a nonmanual social class and to drink 15 U/wk of alcohol or more and less likely to smoke. They had lower prevalence of chronic obstructive pulmonary disease (COPD) and diabetes mellitus; lower mean body mass index (BMI) and waist circumference; lower fasting triglyceride, total cholesterol, CRP, vWF, D-dimer, and NT-proBNP levels; and higher plasma vitamin C, FEV_1_, and high-density lipoprotein cholesterol levels.

**Table 1:** Characteristics of Men According to Physical Activity Score (N = 3,320 Men without Preexisting Coronary Heart Disease, Stroke, or Diabetes Mellitus)

Characteristic	N	None, n = 310	Occasional, n = 764	Light, n = 640	Moderate, n = 514	Moderately Vigorous and Vigorous, n = 1,092	*P* (Trend)
Age	3,320	69.6	68.8	68.5	67.6	67.9	<.001
Manual social class, n (%)	3,113	170 (55)	403 (53)	353 (55)	265 (52)	463 (42)	<.001
>15 U/wk of alcohol, n (%)	3,246	39 (13)	119 (16)	92 (15)	91 (18)	178 (17)	<.001
Current smoker, n (%)	3,316	71 (23)	110 (14)	92 (14)	63 (12)	95 (9)	<.001
Use of antidepressants, n (%)	3,320	16 (5)	26 (3)	14 (2)	13 (3)	25 (2)	.05
Plasma vitamin C, μmol/L, geometric mean	3,090	18.2	20.1	22.2	22.2	24.5	<.001
Body mass index, kg/m^2^, mean^a^	3,311	27.9	27.0	26.6	26.7	26.5	<.001
Waist circumference, cm, mean^a^	3,304	100.7	98.0	96.7	96.7	95.1	<.001
Total cholesterol, mmol/L, mean	3,170	5.9	6.0	6.1	6.1	6.1	.19
High-density lipoprotein cholesterol, mmol/L, mean	3,150	1.3	1.3	1.3	1.3	1.4	<.001
Triglycerides, mmol/L, geometric mean^b^	3,169	0.5	0.5	0.5	0.4	0.4	<.001
Systolic blood pressure, mmHg, mean^a^^,^^b^	3,307	150.4	150.8	150.8	148.3	149.5	.149
Diastolic blood pressure, mmHg, mean^a^^,^^b^	3,307	84.6	85.5	86.4	85.6	85.7	.619
Forced expiratory volume in 1 second, L, mean^a^^,^^c^	3,187	2.0	2.1	2.2	2.4	2.4	<.001
Diabetes mellitus, n (%)	3,320	47 (15)	95 (12)	70 (11)	38 (7)	95 (9)	.001
Chronic obstructive pulmonary disease, n (%)	3,297	86 (28)	212 (28)	181 (28)	107 (21)	236 (22)	<.001
Heart rate, beats/min, mean	3,312	70.0	67.2	66.5	64.6	63.6	<.001
Estimate glomerular filtration rate, mean	3,172	71.9	72.5	73.3	73.9	73.4	.06
Renal dysfunction, n (%)	3,127	47 (16)	94 (13)	65 (11)	48 (10)	102 (10)	.02
C-reactive protein, mg/L, geometric mean	3,186	2.7	1.8	1.6	1.5	1.3	<.001
von Willebrand factor, IU/dL, mean	3,208	151.6	139.2	134.8	135.1	133.5	<.001
D-dimer, ng/mL, geometric mean	3,207	109.9	90.0	81.5	73.7	73.7	<.001
N-terminal pro-brain natriuretic peptide, pg/mL, geometric mean	2,986	121.5	90.0	90.0	81.5	73.7	<.001

a Adjusted for interobserver variation.

b Adjusted for time of day.

c Adjusted for height squared.

### PA and CHD Morbidity and Mortality

The HRs for CHD decreased with increasing PA level (*P* linear trend = .004, Table 2). Compared with men reporting no usual leisure time PA, men reporting occasional, light, moderate, and moderately vigorous or vigorous activity had age- and region-adjusted HRs for first CHD event of 0.52 (95% CI = 0.34–0.79), 0.47 (95% CI = 0.30–0.74), 0.51 (95% CI = 0.32–0.82) and 0.44 (95% CI = 0.29–0.65), respectively. The greatest incremental benefit was from occasional activity. Rerunning models with occasional activity as the reference group did not show evidence of further benefit for more-vigorous activities (data not shown). Adjustment for established behavioral and biological risk factors, including diabetes mellitus, renal function, and depression (Table 2, Model 2), attenuated the associations to a small degree, but the linear trend remained. Further adjustments for CRP, D-dimer, and vWF (Model 3) and NT-proBNP (Model 4) minimally reduced the estimates, and the strong protective effect of PA against CHD events remained, although the linear trend was no longer evident after adjustment for NT-proBNP. Similar patterns were observed for fatal CHD events. The age- and region-adjusted HRs for fatal CHD decreased with increasing PA levels, (*P* for linear trend <.005), (Table 2). For fatal CHD, the greatest incremental benefit was from light activity. Rerunning models with occasional activity as baseline did not show evidence of further benefit from moderately vigorous and vigorous activity (data not shown). Adjustments for CRP, D-dimer, and vWF (Model 3) and NT-proBNP (Model 4) minimally reduced the estimates, and the linear trends were attenuated. The data for all-cause mortality showed a similar pattern to first CHD events, with increasing level of PA strongly protective against mortality. Adjustment for COPD rather than FEV_1_ did not change the pattern of results (data not shown).

**Table 2:** Risk of Coronary Heart Disease (CHD) Mortality in Men According to Physical Activity Score: 1998–2000

Outcome	None, n = 233	Occasional, n = 604	Light, n = 502	Moderate, n = 407	Moderately Vigorous and Vigorous, n = 895	Total, N = 2,641	*P* (Trend)
First CHD event (fatal or nonfatal)							
CHD fatal or nonfatal events, n	37	55	43	35	66	236	
CHD event rate/1,000	18.9	9.5	8.7	8.6	7.4	9.2	
HR (95% CI)							
Model 1	1	0.52 (0.34–0.79)	0.47 (0.30–0.74)	0.51 (0.32–0.82)	0.44 (0.29–0.65)		.004
Model 2	1	0.53 (0.34–0.81)	0.48 (0.31–0.75)	0.55 (0.34–0.89)	0.48 (0.31–0.73)		.02
Model 3	1	0.54 (0.35–0.83)	0.50 (0.32–0.78)	0.57 (0.36–0.93)	0.50 (0.33–0.76)		.04
Model 4	1	0.53 (0.35–0.81)	0.50 (0.32–0.78)	0.57 (0.35–0.92)	0.51 (0.33–0.78)		.05
Fatal CHD							
CHD fatal events, n	21	39	23	19	34	136	
CHD mortality/1,000	10.3	6.6	4.5	4.5	3.7	5.1	
HR (95% CI)							
Model 1	1	0.68 (0.40–1.16)	0.46 (0.26–0.84)	0.55 (0.29–1.02)	0.43 (0.25–0.75)		.005
Model 2	1	0.73 (0.42–1.25)	0.51 (0.28–0.94)	0.67 (0.35–1.27)	0.52 (0.29–0.92)		.04
Model 3	1	0.78 (0.45–1.36)	0.57 (0.31–1.05)	0.74 (0.39–1.41)	0.58 (0.33–1.03)		.09
Model 4	1	0.76 (0.44–1.32)	0.57 (0.31–1.05)	0.73 (0.38–1.39)	0.61 (0.34–1.08)		.14
All-cause mortality							
Deaths, n	110	192	118	88	202	710	
Mortality/1,000	54.1	32.7	23.3	21.1	22.2	27.0	
HR (95% CI)							
Model 1	1	0.61 (0.48–0.77)	0.43 (0.33–0.56)	0.44 (0.33–0.59)	0.45 (0.35–0.57)		<.001
Model 2	1	0.67 (0.53–0.85)	0.49 (0.38–0.65)	0.53 (0.40–0.71)	0.57 (0.45–0.73)		<.001
Model 3	1	0.71 (0.56–0.91)	0.55 (0.42–0.71)	0.58 (0.43–0.78)	0.63 (0.49–0.81)		.003
Model 4	1	0.69 (0.54–0.88)	0.52 (0.40–0.68)	0.56 (0.42–0.75)	0.63 (0.49–0.80)		.004

Model 1 = age and region.

Model 2 = model 1 plus alcohol intake, smoking history, plasma vitamin C, social class, total cholesterol, high-density lipoprotein cholesterol, triglycerides, systolic blood pressure, waist circumference, forced expiratory volume in 1 second, estimated glomerular filtration rate, depression, and diabetes mellitus.

Model 3 = Model 2 plus C-reactive protein, von Willebrand factor, and D-dimer.

Model 4 = Model 2 plus N-terminal pro-brain natriuretic peptide.

HR = Hazard Ratio; CI = Confidence Interval.

### Change in PA Level and CHD Risk

Ninety percent (2,715/3,008) of men included in analyses of PA in 1998 to 2000 had also reported PA levels in a questionnaire 4 years earlier, in 1996; 22% were consistently inactive (did no or only occasional PA) in 1996 and 1998 to 2000, 55% were consistently active (light, moderate, or more-vigorous habitual PA) in 1996 and 1998 to 2000, 14% participated in at least light activity (light, moderate, or more-vigorous PA), and 9% who were active in 1996 had become inactive by 1998 to 2000 (Table 3). Compared to men who were inactive at both times, men who had become active by 1998 to 2000 or were active at both times were younger and more likely to be from a nonmanual social class, drink alcohol regularly, never smoke, not use antidepressants, not have COPD or chronic kidney disease. They were more likely to have higher plasma vitamin C levels; lower BMI, waist circumference and, heart rate; lower triglyceride, CRP, vWF, D-dimer, and NT-proBNP levels; and higher FEV_1_ and eGFR.

**Table 3:** Associations Between Changes in Physical Activity Score Between 1996 and 2000 and Demographic Variables (N = 3,001)

Characteristic	Total, n	Inactive at Both Times	Became Inactive by 1998–2000	Became Active by 1998–2000	Active at Both Times	*P* (Linear Trend)
Participants, n (%)	3,001	680 (22)	274 (9)	411 (14)	1,636 (55)	
Age, mean	3,001	68.7	69.0	67.9	67.9	<.001
Manual social class, n (%)	2,995	342 (51)	149 (54)	231 (56)	728 (45)	<.001
>15 U/wk of alcohol, n (%)	2,929	98 (15)	47 (18)	55 (14)	272 (17)	<.001
Current smoker, n (%)	2,997	109 (16)	40 (15)	53 (13)	159 (10)	<.001
Current use of antidepressants, n (%)	3,001	33 (5)	5 (2)	9 (2)	39 (2)	.005
Vitamin C, μmol/L, geometric mean	2,796	20.49	20.70	22.65	24.05	.001
Body mass index, kg/m^2^[Table-fn tf3-2]	2,994	27.34	27.08	26.87	26.45	<.001
Waist circumference, cm^a^	2,987	99.02	97.84	97.04	95.65	<.001
Total cholesterol, mmol/L	2,866	6.03	6.09	6.05	6.09	.21
High-density lipoprotein cholesterol, mmol/L	2,850	1.30	1.34	1.33	1.35	.00
Triglycerides, mmol/L, geometric mean^b^	2,865	0.54	0.42	0.46	0.45	.01
Systolic blood pressure, mmHg^a^^,^^b^	2,989	150.2	151.0	149.0	149.7	.57
Diastolic blood pressure, mmHg^a^^,^^b^	2,989	85.2	85.2	85.5	86.0	.08
Forced expiratory volume in 1 second, L^a^^,^^c^	2,884	2.12	2.18	2.28	2.37	<.001
Diabetes mellitus, n (%)	3,001	100 (15)	29 (11)	42 (10)	142 (9)	<.001
Chronic obstructive pulmonary disease, n (%)	2,983	187 (28)	76 (28)	113 (28)	365 (22)	.007
Heart rate, beats/min	2,993	67.9	68.1	65.3	64.5	<.001
Estimated glomerular filtration rate	2,868	72.4	72.3	72.7	73.5	.03
Chronic kidney disease, n (%)	2,825	89 (14)	31 (12)	49 (13)	146 (9)	.01
C-reactive protein, mg/L, geometric mean	2,882	2.10	1.93	1.67	1.39	<.001
von Willebrand factor, IU/dL	2,903	142.3	139.7	139.7	132.2	<.001
D-dimer, ng/mL, geometric mean	2,902	90.0	90.9	77.5	73.0	<.001
N-terminal pro-brain natriuretic peptide, pg/mL, geometric mean	2,705	90.9	99.5	84.8	76.7	<.001

Inactive = no or occasional usual physical activity; active = light, moderate, moderately vigorous, or vigorous physical activity, reported at 1996 and 1998 to 2000.

a Adjusted for interobserver variation.

b Adjusted for time of day.

c Adjusted for height squared.

Compared with the baseline group (inactive at both times), HRs for CHD events for men who became inactive were similar to HRs for CHD events for those who remained inactive (HR = 0.87, 95% CI = 0.53–1.45), and likewise for men who became active (HR = 0.86, 95% CI = 0.55–1.35), there was a trend toward protection for men who remained active at both times (HR = 0.73, 95% CI = 0.53–1.02) (Table 4). Equivalent values for fatal CHD were 0.58 (95% CI = 0.29–1.17), 0.67 (95% CI = 0.37–1.21), and 0.58 (95% CI = 0.38–0.89), respectively. Adjustment for established risk factors (Model 2) attenuated the HR for remaining active compared with remaining inactive, but the direction of the estimate toward lower risk remained evident even with adjustments for CRP, D-dimer, and vWF (Model 3) or NT-proBNP (Model 4). For all-cause mortality the HR for events in men who became less active was similar to the HR for events of men who remained inactive. There was a protective effect in men who became more active (HR = 0.65, 95% CI = 0.50–0.85) and in men who remained consistently active (HR = 0.62, 95% CI = 0.51–0.74), adjusted for age and region, which was robust to adjustment in Model 2 and was little attenuated further by addition of CRP, vWF, and D-dimer (Model 3) or NT-proBNP (Model 4). Adjustment for COPD rather than FEV_1_ did not change the pattern of results (data not shown).

**Table 4:** Risk of Coronary Heart Disease (CHD) Mortality in Men According to Change in Physical Activity Score Between 1996 and 2000

Outcome	Inactive at Both Times, n = 525	Became Inactive by 1998–2000, n = 221	Became Active by 1998–2000, n = 331	Active at Both Times, n = 1,316	Total, N = 2,393
First CHD event (fatal or nonfatal)					
CHD fatal or nonfatal events, n	54	21	30	101	206
CHD event rate/1,000	11.0	10.1	9.3	7.7	8.8
HR (95% CI)					
Model 1	1	0.87 (0.53–1.45)	0.86 (0.55–1.35)	0.73 (0.53–1.02)	
Model 2	1	0.84 (0.51–1.41)	0.90 (0.57–1.42)	0.78 (0.55–1.09)	
Model 3	1	0.84 (0.50–1.41)	0.92 (0.58–1.44)	0.79 (0.56–1.12)	
Model 4	1	0.87 (0.52–1.46)	0.92 (0.59–1.45)	0.81 (0.58–1.15)	
Fatal CHD					
CHD fatal events, n	36	10	16	52	114
CHD mortality/1,000	7.2	4.7	4.8	3.9	4.8
HR (95% CI)					
Model 1	1	0.58 (0.29–1.17)	0.67 (0.37–1.21)	0.58 (0.38–0.89)	
Model 2	1	0.56 (0.27–1.15)	0.71 (0.39–1.29)	0.66 (0.42–1.03)	
Model 3	1	0.57 (0.28–1.16)	0.75 (0.41–1.37)	0.71 (0.45–1.11)	
Model 4	1	0.61 (0.30–1.25)	0.74 (0.41–1.36)	0.75 (0.48–1.17)	
All-cause mortality					
Deaths, n	185	70	80	295	630
Deaths/1,000	37.2	32.7	24.0	22.0	26.4
HR (95% CI)					
Model 1	1	0.81 (0.62–1.07)	0.65 (0.50–0.85)	0.62 (0.51–0.74)	
Model 2	1	0.79 (0.60–1.05)	0.71 (0.54–0.93)	0.70 (0.58–0.85)	
Model 3	1	0.81 (0.61–1.07)	0.74 (0.57–0.97)	0.75 (0.61–0.90)	
Model 4	1	0.82 (0.62–1.09)	0.73 (0.56–0.96)	0.75 (0.62–0.91)	

Model 1 = age and region.

Model 2 = Model 1 plus alcohol intake, smoking history, plasma vitamin C, social class, total cholesterol, high-density lipoprotein cholesterol, triglycerides, systolic blood pressure, waist circumference, forced expiratory volume in 1 second, estimated glomerular filtration rate, depression, and diabetes mellitus.

Model 3 = Model 2 plus C-reactive protein, von Willebrand factor, and D-dimer.

Model 4 = Model 2 plus N-terminal pro-brain natriuretic peptide.

HR = Hazard Ratio; CI = Confidence Interval.

In sensitivity analyses, conclusions were not altered when the first 2 years of follow-up were excluded. If the 30.1% of men performing no or occasional activity were light or more-active, the population attributable risk percentage would be a 14% reduction in CHD deaths and 13% in all-cause mortality.

## Discussion

This population-based study of healthy older men found that men who reported even light PA in later life had an approximately 50% lower risk of fatal or nonfatal CHD events than inactive men. There was little evidence of further protection from more-intense PA levels. There was a tendency for lower risk of fatal CHD in men who increased their habitual PA levels from occasional to at least light activity and clearer evidence of substantially lower risk of fatal CHD in those who were active (light or more intense activity) at both times than in those who remained inactive in later life (between the ages of late 50s and 70s). Associations between PA and CHD were robust to adjustment for important established demographic, behavioral, and biological risk factors. There was little evidence that novel biomarkers of inflammation and fibrinolytic activity or cardiac injury were important mediators of the association between PA and nonfatal CHD, although there was some evidence that they mediated the association between PA and fatal CHD events and all-cause mortality. This study is one of few prospective cohort studies relating PA in later life and change in PA to CHD risk in older adults. It extends prior findings from largely middle-aged population samples into an older age group and adds new information about mediating pathways between PA and CHD risk.

### PA at One Time Point in Later Life: Relationship to CHD Risk

Most evidence about the shape of the dose-response curve between PA and CHD relates to middle-aged rather than older adults.^2^ In the current sample of older adults, potentially beneficial effects of even occasional and light PA on risk of fatal or nonfatal CHD were found and that the greatest incremental benefits may accrue from light activity. Occasional PA in the current study included regular walking and some recreational activity but not vigorous sporting activity. The results fit with recent evidence that lighter activities are protective against CHD, suggesting that some activity is better than none.^2^ Light activity or more was associated with approximately half the risk of CHD mortality and morbidity, which was estimated as greater than 30% to 35% in a systematic review largely using data from middle-aged adults.^2^ Associations with nonfatal CHD were of similar magnitude to those reported in the British Regional Heart Study cohort when they were 20 years younger, although the benefits from moderate and moderate to vigorous activity may be somewhat stronger in middle age.^19^ Regular walking has been associated with halving the risk of CHD events in elderly men^21^ and women,^22^ and 40% less CVD mortality was reported for older adults performing more than 30 minutes of activity per day (including low-intensity stretching exercises and walking slowly) than for those performing none.^23^ PA has substantial potential to reduce avoidable CHD deaths in elderly adults; in the current sample, if those who were less active became at least light active (regular daily walking and some recreational activity, e.g., gardening, yard work, household chores), a 14% reduction in CHD mortality would be expected.

### Changes in PA in Later Life: Relationship to CHD Risk

In the present study, men who remained active and those who became active had lower CHD risk (particularly for fatal events) than those who remained or became inactive. A protective effect of increasing PA level in older age on all-cause or CHD mortality is reported in a few studies of general populations,^24^–^27^although not all report conclusive findings.^28^ An investigation of this cohort when younger found that increasing PA levels during middle age or maintaining at least light activity was associated with lower all-cause and CVD mortality than persistently low activity levels.^29^

### Pathways

The association between PA and fatal CHD operated through established risk factors, consistent with expectations from studies of middle-aged adults,^19^ but their explanatory power was small, suggesting that PA affects CHD risk in older adults by other means. Previous studies suggest that inflammatory markers may play an important role in mediating the effects of PA on CHD in middle-aged populations,^13^,^14^,^30^ and the current study found that adjustment for CRP, vWF, and D-dimer (which have previously been linked to CHD risk^4^,^9^–^11^,^31^ and PA levels^5^,^32^) partially explained associations between PA and CHD. NT-proBNP was included to indicate presence of cardiac injury and level of cardiac function. Some evidence was found that NT-proBNP may be important in explaining the onset of fatal CHD events, which fits with other evidence linking NT-proBNP more strongly to CHD mortality than morbidity.^6^

### Strengths and Limitations

This study benefits from prospective data with high follow-up rates, validated MI events, and a PA score validated against biological measures (e.g., FEV_1_ and heart rate^5^,^19^). The mediating role of a wide range of established and novel biological risk factors that may be on the causal pathway between activity and CHD risk was investigated. The possibility of residual confounding cannot be excluded, but many important behavioral and social confounders were adjusted for. To reduce risks of reverse causality, men with preexisting CHD, stroke, or heart failure or limited mobility were excluded because they have high risk of mortality and are likely to limit their PA because of their prior health, and the first 2 years of follow-up were excluded in a sensitivity analysis. The data about PA at more than one time point in later life enabled investigation of how changes in PA were related to incidence of CHD, although small numbers of CHD events in one of the change groups were a limitation. Data were lacking on cardiorespiratory and muscular fitness, which are shown to be related to onset of CVD and to CVD risk factors.^33^–^35^ The current study was limited to healthy men, so results cannot be generalized to older women or those with preexisting CVD, although the sample was socioeconomically representative of older men in the United Kingdom and had exceptionally high follow-up rates.

In an observational study, it is not possible to establish causality of the associations observed between PA and CHD, but men with prevalent CHD, stroke, and heart failure were excluded to minimize the chance that any findings were due to men reducing their PA levels because of presence of disease (reverse causality). The analyses were adjusted for a wide range of confounding factors, and data on PA at more than one time point in later life were also included, which enabled how changes in PA were related to incidence of CHD to be examined, which gives a stronger level of evidence than using PA from just one time point. Finally, the findings suggesting that PA may protect against onset of CHD morbidity and total mortality fit with a large body of research (clinical and observational) that suggests that PA protects against onset of CHD.^36^

## Conclusion

PA in later life is potentially important in primary prevention of CHD and all-cause mortality in older men. Even very modest levels of PA were associated with approximately half the risk of CHD morbidity and all-cause mortality, in part mediated by the beneficial effects on adiposity and blood lipids, inflammatory and hemostatic mechanisms, and cardiac injury (NT-proBNP). Taking up PA in later life was associated with approximately one-third lower risk of all-cause mortality, although strong evidence of reduction in CHD events was not found (although numbers were low). There was some evidence that remaining active in later life protected against CHD and all-cause mortality. Promotion of PA in later life is therefore important. Preventing the age-related declines in PA levels observed in many populations could markedly reduce CHD risk in older adults, who are a high-risk group.
